# Amphiphilicity-Controlled Localization of Red Emitting Bicationic Fluorophores in Tumor Cells Acting as Bio-Probes and Anticancer Drugs

**DOI:** 10.3390/molecules27123713

**Published:** 2022-06-09

**Authors:** Alessio Cesaretti, Letizia Mencaroni, Carmela Bonaccorso, Valentina Botti, Eleonora Calzoni, Benedetta Carlotti, Cosimo Gianluca Fortuna, Nicolò Montegiove, Anna Spalletti, Fausto Elisei

**Affiliations:** 1 Department of Chemistry, Biology and Biotechnology and “Centro di Eccellenza Materiali Innovativi Nanostrutturati” (CEMIN), University of Perugia, Via Elce di Sotto 8, 06123 Perugia, Italy; alex.cesaretti14@gmail.com (A.C.); valentinabotti.90@hotmail.it (V.B.); eleonoracalzoni@gmail.com (E.C.); benedetta.carlotti@unipg.it (B.C.); nicolo.montegiove@gmail.com (N.M.); anna.spalletti@unipg.it (A.S.); fausto.elisei@unipg.it (F.E.); 2 Department of Chemical Sciences, University of Catania, Viale Andrea Doria 6, 95125 Catania, Italy; carmela.bonaccorso@unict.it (C.B.); cg.fortuna@unict.it (C.G.F.)

**Keywords:** bications, far-red emission, ultrafast transient absorption spectroscopy, nucleic acid binders, antiproliferative effect, cell-permeant dyes, lipophilicity, bioimaging

## Abstract

Small organic molecules arouse lively interest for their plethora of possible biological applications, such as anticancer therapy, for their ability to interact with nucleic acids, or bioimaging, thanks to their fluorescence emission. Here, a panchromatic series of styryl-azinium bicationic dyes, which have already proved to exhibit high water-solubility and significant red fluorescence in water, were investigated through spectrofluorimetric titrations to assess the extent of their association constants with DNA and RNA. Femtosecond-resolved transient absorption spectroscopy was also employed to characterize the changes in the photophysical properties of these fluorophores upon interaction with their biological targets. Finally, in vitro experiments conducted on tumor cell lines revealed that some of the bicationic fluorophores had a peculiar localization within cell nuclei exerting important antiproliferative effects, others were instead found to localize in the cytoplasm without leading to cell death, being useful to mark specific organelles in light of live cell bioimaging. Interestingly, this molecule-dependent behavior matched the different amphiphilicity featured by these bioactive compounds, which are thus expected to be caught in a tug-of-war between lipophilicity, ensured by the presence of aromatic rings and needed to pass cell membranes, and hydrophilicity, granted by charged groups and necessary for stability in aqueous media.

## 1. Introduction

Small organic fluorescent molecules are pivotal for a myriad of research applications ranging from optoelectronics [1,2], light-harvesting [3], sensing [4,5,6,7,8,9], to the medical and biological fields, particularly for bioimaging [10,11,12], disease diagnostics and cancer therapeutics [13,14]. Jointly to long-wavelength emission, which guarantees deeper penetration in biological tissues [15], other properties such as aromaticity, flexibility, photostability in physiological environments, lipophilicity, and solubility in an aqueous medium are sought-after features for designing bioavailable probes and drug-like molecules [16,17,18,19]. So far, many well-known fluorophores (quinoline, styrene, xanthone, coumarin, fluorescein, rhodamine, BODIPY, naphthalimide, and cyanine) have been taken into consideration as parent structures by virtue of their emissive capability and consequently chemically-tailored to obtain bioprobes [20]. The peculiar and rapid optical response, accounting for the changes in the photochemical properties of the markers when selectively bound to their cellular biotargets in specific intracellular compartments [14,21], is a powerful tool for either monitoring living cells and organelles or performing therapeutic action when dealing with cytotoxicity [22]. However, many of these fluorescent derivatives cannot find practical applications due to their limited biocompatibility. In this regard, the bottleneck is usually the poor water solubility of small organic fluorophores but also their inefficient cellular permeability. For these reasons, there is still the need to identify and develop ideal bioactive compounds.

Jun et al. have recently rationalized some key rules for designing small organic fluorophores with long-wavelength emission properties suitable for biological applications [23]. They stated that: (i) a push–pull character, granted by the presence of electron-donor (D) and electron acceptor (A) groups arranged in an A―π―D-like structure, is ideal for increasing the intramolecular charge transfer (ICT), which implies a bathochromic shift of both the absorption and emission spectra; (ii) an extended and conjugated molecular structure contributes to the reddening of the spectra and the increase of the absorption coefficient; and (iii) the introduction of rotating substituent can positively affect the Stokes shift. Besides this rational design, numerous former studies pointed out that cationic styryl dyes manifest good affinities for distinct types of DNAs and RNAs [24,25,26,27,28,29,30,31], and also alluring anticancer effects against different cell lines [32,33,34,35].

**Scheme 1:**
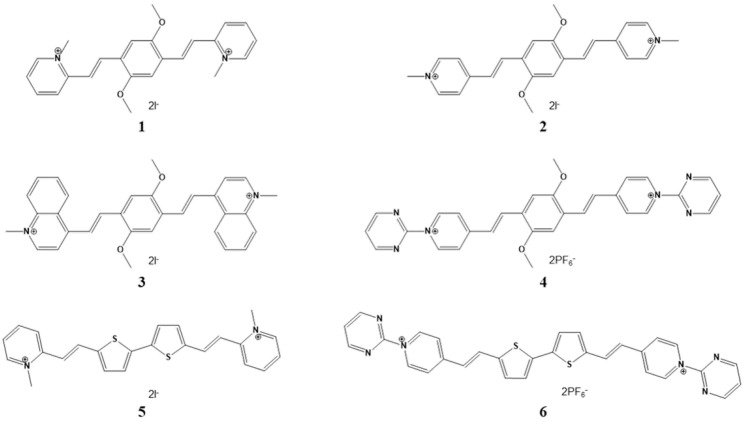
Investigated compounds.

Within this broader context, we have recently synthesized a series of six quadrupolar styryl azinium dyes, having a more extended A^+^―π―D―π―A^+^ structure (Scheme 1). These bicationic fluorophores were thoroughly characterized as for their photobehavior in solvents of different polarity and their nonlinear optical response [36]. Interestingly, the net positive charges in these double-arm push–pull molecules provided enhanced ICT character, resulting in notable two-photon absorption (TPA) cross-sections, and improved water solubility. The significant fluorescence capability shown in aqueous solution by these bicationic compounds, which can thus be excited in the biological window by means of TPA, their conjugated flexible structures, large Stokes shift and absorption coefficients, and their absorption and fluorescence spectra spanning from green to far-red highlighted the potential use of this panchromatic series of dyes in biological applications. Here, aiming at testing the actual biocompatibility of the six bications, the binding affinity with nucleic acids, namely tRNA and ct-DNA, was checked through spectrophotometric and fluorimetric titrations. A deeper insight into the photophysical properties of the emissive bication/target complexes was achieved with the aid of femtosecond transient absorption spectroscopy. Further to this, the antiproliferative effect exerted by the six molecules on two tumor cell lines, namely human alveolar basal epithelial (A549) and cervical epithelial (HeLa) adenocarcinoma cells, was assessed through the MTT assay, and the results were interpreted in terms of specific localization in different cellular compartments, as revealed by fluorescence microscopy. In particular, the peculiar amphiphilicity of each bication was identified as the key feature for guiding the fluorophores within the cells, making them promising candidates as long-wavelength emissive dyes for bioimaging and/or active anticancer drugs. 

## 2. Results

### 2.1. Spectrophotometric and Fluorimetric Investigation of the Interaction with Nucleic Acids

**Figure 1:**
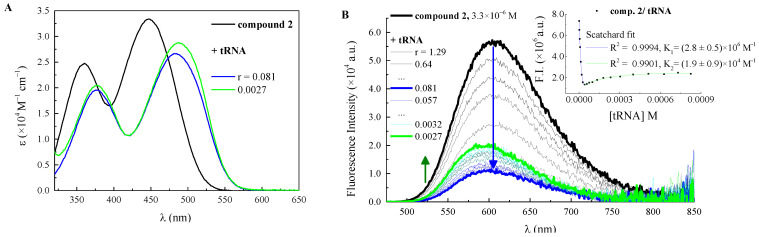
(**A**) quantitative absorption spectra of **2** and its complexes with tRNA in ETN buffer solution, pH 7.4, with r = [compound]/[nucleic acid]. (**B**) changes in the fluorescence spectrum of **2** (λ_exc_ = 448 nm, c = 3.3 µM) in ETN buffer solution, pH 7.4, upon titration with tRNA; Inset: fluorescence intensity of **2** as a function of tRNA concentration and its fitting according to the Scatchard equation (n = 0.2).

**Figure 2:**
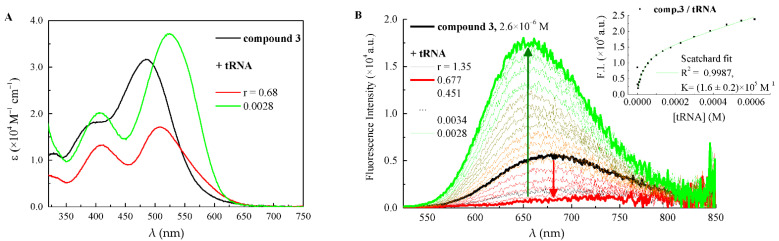
(**A**) qantitative absorption spectra of **3** and its complexes with tRNA in ETN buffer solution, pH 7.4, with r = [compound]/[nucleic acid]. (**B**) changes in the fluorescence spectrum of **3** (λ_exc_ = 485 nm, c = 2.6 µM) in ETN buffer solution, pH 7.4, upon titration with tRNA; Inset: fluorescence intensity of **3** as a function of tRNA concentration and its fitting according to the Scatchard equation (n = 0.2).

**Figure 3:**
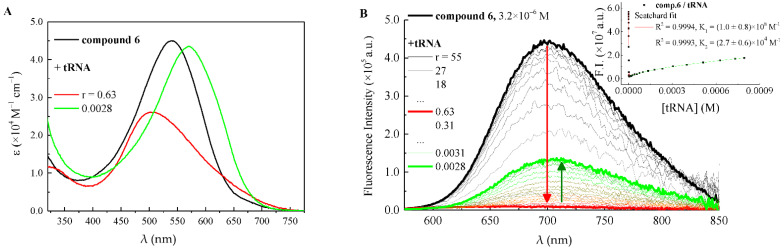
(**A**) quantitative absorption spectra of **6** and its complexes with tRNA in ETN buffer solution, pH 7.4, with r = [compound]/[nucleic acid]. (**B**) changes in the fluorescence spectrum of **6** (λ_exc_ = 540 nm, c = 3.2 µM) in ETN buffer solution, pH 7.4, upon titration with tRNA; Inset: fluorescence intensity of **6** as a function of tRNA concentration and its fitting according to the Scatchard equation (first equilibrium n = 1; second equilibrium n = 0.2).

The interaction of the six bications under investigation with nucleic acids, i.e., tRNA and ct-DNA, was evaluated by means of spectrophotometric and fluorimetric titrations carried out in ETN buffer, pH 7.4. Complexations of the six compounds with their biological targets were studied with ratios r (= [compound]/[nucleic acid]) going from 1.3 to 0.002 in order to assess the expected behavior in a biological environment where nucleic acids would be in excess relative to the fluorophores. All of the molecules showed a great affinity with their biological targets, as highlighted by significant modifications of their absorption and emission spectra upon increasing additions of tRNA and ct-DNA (Figure 1, Figure 2, Figure 3 and Figures S1–S11 in Supplementary Materials). The spectral and fluorescence properties of the complexes are listed in Table 1. The investigated bications are all characterized by large Stokes shifts, resulting from important intramolecular rearrangement following excitation (charge transfer processes and possibly twisting) [36]; however, these shifts were found to reduce upon complexation indicating that the binding with polynucleotides impaired the freedom of the molecules.

As a rule, the interaction with nucleic acids caused the absorption spectrum to shift bathochromically. This could be the result of both the reduced polarity experienced by these negatively-solvatochromic dyes upon binding with nucleic acids [36], and the π–π interactions among aromatic portions, common to both the investigated compounds and their biological targets. An exception is compound **6** (Figure 3); in fact, its spectrophotometric titration with tRNA revealed an initial blue-shift of the absorption band at high r values, followed by the typical red-shift at higher tRNA concentrations. 

However, in all cases, depending on the r range considered, two distinct changes on the absorption spectrum can be recognized: the first features a marked hypochromic effect while the second is characterized by an increase in absorbance. The former is detectable at relatively low concentrations of nucleic acid (r > 0.10) and could reasonably be assigned to a type of interaction that is mainly driven by electrostatic attraction between the bicationic molecules and the negatively charged phosphate backbone of polynucleotide chains. On the other hand, the latter, featured in a large excess of tRNA and ct-DNA, shows absorbance enhancement and further red-shift, likely related to the increased planarity and conjugation taken upon complexation, peculiar to a more specific binding with the nucleic acids, supposedly intercalation between adjacent bases or groove binding.

As for the fluorimetric titrations, different trends emerged for the specific bication-nucleic acid pair considered. All the titrations are reported in Figure 1, Figure 2, Figure 3 and Figures S7–S11, while the binding constants (logK), calculated by processing fluorimetric titrations according to the Scatchard equation (McGhee–von Hippel formalism) [37], are reported in Table 2. In the case of compounds **1** and **2**, which are highly fluorescent molecules in aqueous solution (Φ_F_ = 0.16 and 0.36, respectively) [36], their fluorescence was found to undergo a significant decrease in the presence of tRNA (Figure 1 and Figure S7). In fact, their emission properties upon additions of ct-DNA and synthetic polynucleotides of defined composition had previously been studied and a fluorescence drop had already been revealed [24]. When investigating their behavior in the presence of tRNA, **1** experienced a monotonous and sharp decrease of fluorescence (the fluorescence quantum efficiency of **1**-tRNA complex, Φ_F/tRNA complex_, reduced to 0.025), while **2** featured an initial significant drop (Φ_F/tRNA complex1_ = 0.066) up to r = 0.081, followed by a moderate increase (Φ_F/tRNA complex2_ = 0.12) at higher tRNA concentrations. The fluorimetric titrations allowed the binding constants to be derived through the Scatchard equation (Table 2) by fixing the ratio n (= [bound compound]/[polynucleotide phosphates]) to the value of 0.2: the association of **1** with tRNA features an association constant logarithm (logK) of 5.49 ± 0.06, significantly smaller than the binding constant previously measured for ct-DNA (logK = 7.11 ± 0.04) [24]. On the other hand, the double trend recorded for **2** in the presence of tRNA allowed two constants to be measured, both with an n value of 0.2 and thus assigned to two distinct specific binding modes with this nucleic acid: the first equilibrium is described by a value of logK = 6.4 ± 0.2, comparable to the logK previously found with ct-DNA (logK = 6.85 ± 0.04) [24], while the second displays a smaller value of 4.3 ± 0.5.

When it comes to the other compounds, the first additions of polynucleotides (up to r = 0.30 depending on the molecule) caused either no important modifications or dramatic quenching of the fluorescence efficiency, while higher amounts of both tRNA and ct-DNA were responsible for a significant emission enhancement (Figure 2, Figure 3 and Figures S8–S11). With a view to the potential interest for these compounds in biological applications, the complexation taking place at low r values (excess of polynucleotides) is the one deserving special attention, both for its higher fluorescence and its expected occurrence in a cellular environment. However, in the particular case of **6** with tRNA, for which the first interaction was found to shift hypsochromically the absorption band, the initial binding mechanism was also scrutinized (Figure 3). Specifically, tRNA additions from r = 55 to r = 0.63 brought about a dramatic drop of the emission efficiency (Φ_F_ = 0.058 → Φ_F/tRNA complex1_ = 0.002), while the following interaction restored the fluorescence to about 30% of the initial value Φ_F/tRNA complex2_ = 0.018 when r = 0.0028. Each part of the double trend found for the emission efficiency of **6** as a function of tRNA concentration was fitted by the Scatchard equation to obtain the binding constants for the two equilibria. Interestingly enough, while it was possible to fit the second association event (logK = 4.4 ± 0.2) with the ratio n = 0.2, as it is typical of specific complexation by intercalation or groove binding [38], the first complexation (logK = 8.0 ± 0.8) required n = 1, involving an interaction in a 1:1 ratio between **6** and tRNA phosphates. This result could suggest the agglomeration of molecules along the polynucleotide helix implying that electrostatic attraction guides the initial interaction between bications and polynucleotides [39]. By looking at Table 1, it is apparent that, for all compounds **3–6**, the complexation with tRNA leads to reduced fluorescence efficiency relative to ct-DNA. It is important to highlight that these bications become upon complexation better fluorophores than the isolated molecules in solution, with the exclusion of **6** with tRNA, whose complex is less emissive than the free ligand. On the other hand, its complex with ct-DNA features the highest fluorescence efficiency, which becomes the main deactivation process for the bound molecule (Φ_F/ct-DNA complex_ = 0.56).

The binding constant values for the biologically-interesting complexation occurring in excess of nucleic acids (and thus anticipated under biological conditions) are significant for the whole bicationic series (of the order of logK = 4–7). The constants describing the interaction with ct-DNA are generally higher than or comparable to those with tRNA, except for compound **3**, which was found to have a slightly greater affinity for the tRNA molecule (logK_tRNA_ = 5.2 ± 0.1) relative to the ct-DNA (logK_ct-DNA_ = 4.66 ± 0.09). 

### 2.2. Femtosecond Transient Absorption Spectroscopy of Complexes with Nucleic Acids

**Figure 4:**
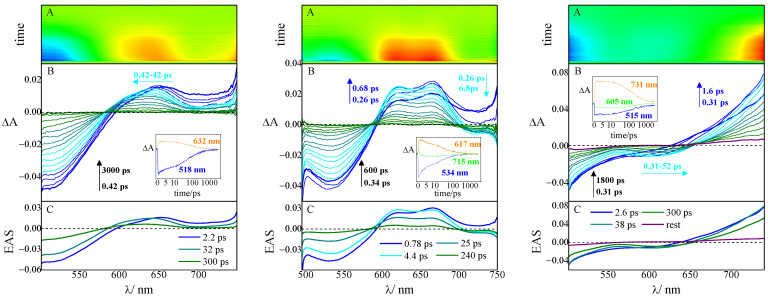
Pump–probe absorption spectroscopy of compound **3** (**left graph**), **4** (**central graph**), and **5** (**right graph**) in ETN buffer solution, pH 7.4, complexed with tRNA, r ([compound]/[tRNA]) = 0.005 (λ_exc_ = 400 nm): (**A**) contour plot of the experimental data, (**B**) time-resolved absorption spectra recorded at different delays after the laser pulse (inset: decay kinetics recorded at meaningful wavelengths), and (**C**) Evolution Associated Spectra (EAS) obtained by global analysis.

**Figure 5:**
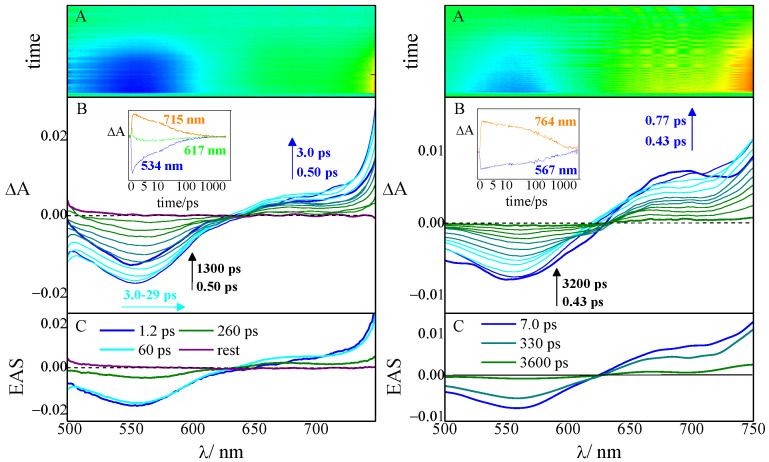
Pump–probe absorption spectroscopy of compound **6** in ETN buffer solution, pH 7.4, complexed with tRNA (**left graph**) and ct-DNA (**right graph**), r ([compound]/[nucleic acid]) = 0.005 (λ_exc_ = 400 nm): (**A**) contour plot of the experimental data, (**B**) time-resolved absorption spectra recorded at different delays after the laser pulse (inset: decay kinetics recorded at meaningful wavelengths), and (**C**) Evolution Associated Spectra (EAS) obtained by global analysis.

With an eye to biological applications, the complexes obtained in a large excess of polynucleotides were also characterized for their excited-state deactivation properties by means of femtosecond-resolved transient absorption (TA) spectroscopy. Measurements were carried out at r ([compound]/[nucleic acid]) = 0.005 in order to ensure that most of the bication ligand would be in the bound form. Figure 4, Figure 5 and Figures S12 and S13 show the data matrix (upper panel: colored map, ΔA vs. λ and τ, on *x*-axis and *y*-axis, respectively), representative spectra and kinetics (central panel), and Evolution Associated Spectra (EAS) of the transients describing the excited-state dynamics with their characteristic lifetimes (lower panel) [40]. Table 3 compiles the results of the global fitting analysis carried out by GloTarAn software [41] together with the excited-state properties of the free molecules in water [36], reported for comparison purposes. 

As for compounds **1** and **2**, the excited-state lifetimes for the S_1_ state of their complexes with tRNA are shorter than those of the free ligands (τ**_1_**_+tRNA_ = 1600 ps vs. τ**_1_**_,free_ = 2700 ps and τ**_2_**_+tRNA_ = 1200 ps vs. τ**_2_**_,free_ = 1500 ps), in line with the marked fluorescent quenching recorded upon complexation. In contrast, the ct-DNA complexes were found to show longer lifetimes (τ**_1_**_+ct-DNA_ = 3500 ps and τ**_2_**_+ct-DNA_ = 4500 ps) probably by virtue of their interaction with AT-base pairs, which had been found in a previous study to cause a minor reduction (in the case of **1**) or an enhancement (in the case of **2**) in the emission efficiency of these compounds [24]. However, the excited-state dynamics recorded at early delays are also described by a number of shorter transients that can be assigned to either distinct relaxation phenomena (including solvation, Solv., vibrational relaxation, VR, and conformational relaxation, CR) depending on their specific lifetimes, or states ascribable to ligand–polynucleotide interactions. In particular, owing to their peculiar spectral profiles and characteristic lifetimes, the same short transients that had previously been detected in the presence of ct-DNA [24] can again be recognized upon complexation with tRNA (Figure S12).

In the case of compounds **3–5**, the progression of the excited states follows similar dynamics and spectral evolution when either the complexes with tRNA or those with ct-DNA are considered (the results in the presence of tRNA are reported in Figure 4). Negative signals due to Ground State Bleaching (GSB, blue in the colored map) can be recognized in the short-wavelength region, reflecting the peculiar position of the absorption spectrum of the complexes, while positive signals of Excited-State Absorption (ESA, orange in the colored map) are typical of the different molecules. Only in the case of compound **5**, a negative band, centered at about 600 nm, can be identified in the same spectral position of the steady-state fluorescence and thus assigned to the Stimulated Emission (SE, light blue in the colored map) of the complex. The S_1_ lifetimes obtained by the global analysis of the data (Table 3) are significantly longer for the complexes than for the unbound molecules, in line with the fluorescent enhancement acquired upon complexation, as a result of reduced freedom of molecular motions. In particular, the complexes with ct-DNA always feature slightly longer lifetimes than those with tRNA. 

The excited-state dynamics of the complexes of compound **6** (Figure 5) reflect the differences that emerged from the fluorimetric titrations. Independently of the polynucleotide considered, the spectral evolution is characterized by a negative band of GSB, centered at about 560 nm and matching the absorption maximum of the complexes, and the onset of an ESA band in the red part of the spectrum; however, the temporal evolution is quite different with a faster dynamic peculiar to the tRNA complex and a much slower dynamic characteristic of the ct-DNA complex. The outcomes of the fitting (Table 3) revealed that the lifetime of the former is indeed shorter (τ**_6_**_+tRNA_ = 260 ps) when compared with both the ct-DNA complex (τ**_6_**_+ct-DNA_ = 3600 ps) and the unbound ligand (τ**_6_**_,free_ = 910 ps), in a parallel trend with the fluorescence efficiencies. In fact, the fluorescent constant for the emissive state is unchanged when compound **6** binds tRNA (k_F**6**,free_ = 0.64 × 10^8^ s^−1^ and k_F**6**+tRNA_ = 0.69 × 10^8^ s^−1^), while it doubles when **6** complexes ct-DNA (k_F**6**+ct-DNA_ = 1.6 × 10^8^ s^−1^).

### 2.3. Antiproliferative Effect and Cellular Localization in Tumor Cells 

Once the interaction of the six bications with their biological targets had been characterized and their complexes described, biological experiments were carried out in order to understand the potentiality of this class of molecules for biological applications. The latter can be distinguished in bioimaging applications as fluorescent markers, by exploiting the emission properties of the bound ligands, and therapeutic applications as anticancer drugs, by resorting to the cytotoxic effect that small organic molecules can exert upon binding with nucleic acids. 

The antiproliferative activity of compounds **1–6** against two tumor cell lines, human alveolar basal epithelial (A549) and cervical epithelial (HeLa) adenocarcinoma cells, was thus tested by the MTT assay in the 10^−4^–10^−8^ M concentration range, and the antiproliferative effect was estimated by measuring the IC_50_ index, which provides for the concentration responsible for 50% growth inhibition after 72-h incubation (Figure S14 and Table 4). The analysis showed that the six molecules had distinct activities between one another and different behaviors against the two cell lines. In particular, compound **1** did not exhibit any effect on cell viability in both cases in all the range of concentrations explored. Compound **4** was found not to alter the vitality of A549 cells, while it was fairly damaging to the metabolism of HeLa cells (IC_50_ = 13 µM), and compound **2** showed clear inhibition of cell growth only at concentrations higher than 10 µM, with an IC_50_ value of 53 µM for HeLa cells and an estimated value > 100 µM against A549 cells. The other three compounds (**3**, **5,** and **6**) can instead be regarded as anticancer drugs independently of the cell line considered, with the highest antiproliferative effect observed against HeLa cells, particularly in the case of **6**, whose IC_50_ reaches a sub-micromolar value of just 0.7 µM.

**Figure 6:**
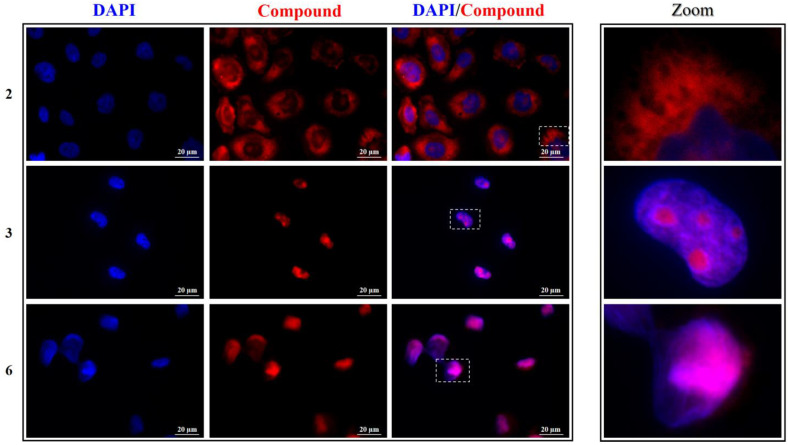
**Left panel**: representative fluorescence microscopy images of nuclei (DAPI, blue), compounds **2**, **3**, and **6** (red), and the relative merged images of HeLa cells. Image magnification: 60×. **Right panel**: zoom-in of representative cells.

**Figure 7:**
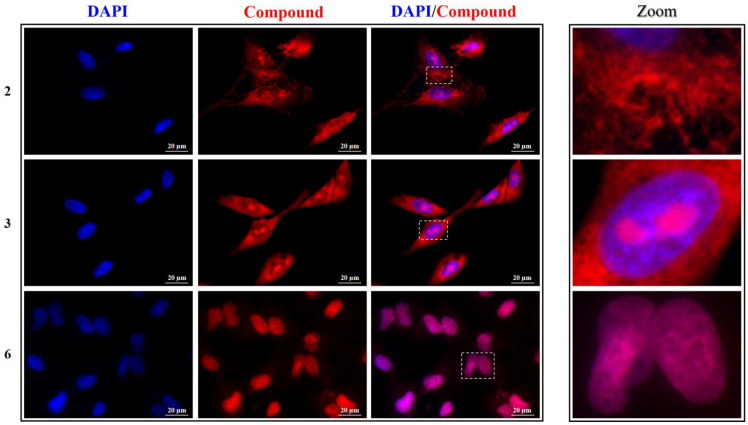
**Left panel**: representative fluorescence microscopy images of nuclei (DAPI, blue), compounds **2**, **3**, and **6** (red), and the relative merged images of A549 cells. Image magnification: 60×. **Right panel**: zoom-in of representative cells.

With the aim of correlating the antiproliferative effect with the localization of the dyes within cells, fluorescence microscopy images of the two tumor cell lines were taken after 2-h incubation with a 10 µM concentration of the six bications, exploiting their significant emission upon binding with nucleic acids. Cells were also stained with the nuclear dye DAPI in order to visualize cell nuclei. Images of compounds **2**, **3**, and **6** in HeLa and A549 cells are shown in Figure 6 and Figure 7, respectively, while the other images are reported in Figures S15 and S16. No photobleaching was observed upon irradiation up to 5 min. All the six molecules were found to penetrate and stain efficiently the cells, but their localization was different depending on the specific compound/cell combination. Specifically, those molecules able to reach the cell nuclei were those which also exhibited the strongest antiproliferative effect (**3**, **5**, and **6**). In fact, the green fluorescence of compound **1** can be seen diffused in all the cytoplasm for both cell lines with only a partial localization in the perinuclear region, without staining the cell nuclei, consistently with its non-toxic effect. The behavior of compound **4** was instead different depending on the cell type considered: when dealing with A549, **4** exhibits a homogeneous distribution within the cell, while in the HeLa cells, its red fluorescence is mainly localized in the nuclei with a good overlap with DAPI’s blue emission. This finding parallels the results of the MTT assay, revealing that compound **4** can act as an anticancer drug only against HeLa cells. 

Compound **2** showed a mild antiproliferative activity and indeed its fluorescence is mainly found around the nuclei, but not homogeneously distributed in the cytoplasm: this compound seems to specifically stain distinct cellular structures that could be associated with the endoplasmic reticulum. This might result in the interference of **2** in the normal metabolism of the cells, thus affecting their vitality to some extent, inasmuch as various functions take place in this endomembrane system, e.g., protein synthesis thanks to the associated ribosomes, protein co- and post-translational modifications, and transport of translated proteins to the Golgi apparatus [42]. However, this behavior deserves much more attention and will be the object of further future studies. Compound **3** exhibited a marked antiproliferative effect and, in fact, not only does it enter the cell nuclei, but it specifically stains particular nuclear regions recognized as the nucleoli. The latter are visualized as black dots in the DAPI images, since DNA, the target of DAPI, has a threefold lower concentration in the nucleolus compared to the surrounding nucleoplasm [43]. Nucleoli are instead rich in RNA, and compound **3** was indeed found to have a greater affinity for RNA than DNA. This especially applies to HeLa cells (Figure 6), where **3** is exclusively observed as bright dots in the nucleoli, coherently with its low IC_50_ concentration. This localization is still recognizable in A549 cells, but in this case, an important fluorescence is also seen diffused in the cytoplasm (Figure 7).

Finally, compounds **5** and **6**, which proved to act as antiproliferative compounds against both cell lines, were able to effectively enter the nuclei (Figure 6, Figure 7 and Figures S15 and S16) and stain them with a pattern that overlaps entirely that of DAPI. This result is corroborated by the greater affinity of these two molecules for DNA and the larger fluorescent efficiency revealed upon binding with this nucleic acid. Moreover, cell nucleus morphology was affected by both compounds: signs of chromatin condensation and blister formation, which are precursor indicators for nuclear fragmentation in cells undergoing apoptosis [44,45], and can be discerned through the images taken after incubation with the two dyes, implying their plausible direct cytotoxicity.

## 3. Discussion

The series of bicationic dyes under investigation attracts special interest because of their enhanced solubility in aqueous media, as compared to analogous neutral or monocationic compounds, and their extended aromaticity, which anticipates their ability of binding nucleic acids. Another feature peculiar to these compounds is the high conjugation and ICT character responsible for shifting their absorption and emission spectra in the visible, up to the far-red region of the spectrum. The latter is indeed a desirable property in the development of long-wavelength fluorescent dyes for biological applications, in that it allows deeper tissue penetration and reduced background from biomolecule autofluorescence [46,47]. 

In particular, the whole series of dyes displayed a high affinity for both DNA and RNA polynucleotides. The marked changes in the emission properties upon binding allowed association constants in the range logK = 4–7 to be measured from fluorimetric titrations by resorting to the Scatchard equation, based on the McGhee–von Hippel formalism. The latter well correlated with the experimental data by fixing the parameter n = [bound compound]/[polynucleotide phosphates] to 0.2 in agreement with a definite mode of binding, typically intercalation or groove binding, between the dye and the nucleobases [38]. When dealing with compound **6**, the interaction occurring at low tRNA concentration (r > 0.63) was also investigated and found to be described by n = 1, which is likely the sign of non-specific electrostatic interactions that lead to agglomeration of the bication along the polynucleotide backbone. This implies that electrostatic attraction is the driving force for the initial interaction of the investigated compounds with their biological targets, disclosing the twofold importance of their double positive charge, besides enhancing solubility in aqueous media.

As a rule, the complexation of the dyes with nucleic acids caused their fluorescence efficiency to enhance as a consequence of the confinement of the molecule in a more rigid conformation acquired following the binding to the nucleobases, in a parallel trend with the lengthening of the excited-state lifetime revealed by transient absorption spectroscopy with femtosecond resolution in time. An exception to this behavior is represented by compounds **1** and **2** with both nucleotides and **6** with tRNA, supposedly because of specific interactions.

Biological experiments conducted on two different tumor cell lines revealed a net correlation between the inhibition of cell growth and cellular localization: the more the dye is able to reach the nucleus and be preferentially accumulated within, the stronger the antiproliferative effect. As such, compounds **5** and **6**, entirely localized in the nucleus, are the most promising anticancer bications of the series, showing also distinct signs of cytotoxicity upon binding with nuclear DNA, while **1** does not exhibit any antiproliferative activity because of its inability to penetrate the nuclear membrane. This behavior could be rationalized by taking into account the lipophilicity of these compounds as described by the cLogP (consensus LogP) descriptor (calculated using the SwissADME web tool) [48], where *p* is the value of the 1-octanol/water partition coefficient. In fact, lipophilicity is a key feature that molecules need to possess to come across cellular membranes. A threshold value of cLogP = 1 can discriminate small organic molecules between permeant (cLogP > 1) and impermeant (cLogP < 1) dyes [49,50]. The six bications are all characterized by cLogP values moderately higher than 1 (Table S1), making them good cellular probes able to pass the cellular outer membrane. However, compounds **3**, **5**, and **6** exhibit the greatest cLogP values (cLogP > 4.5), while compounds **1**, **2**, and **4** have cLogP ≤ 3.5. Since the cell membrane is made up of a single lipid bilayer while the nuclear membrane comprises two lipid bilayer structures, this difference is crucial for discriminating molecules that can easily reach the nuclei (**3**, **5**, and **6**) from molecules that are preferentially localized in the cytoplasm (**1**, **2**, and **4**). Hence, small modifications in the molecular structure can significantly alter the amphiphilic character, and thus the bioavailability and bioactivity, of these styryl azinium dyes. A decisive step in this direction is therefore represented by the substitution of the central donor group from the dimethoxy phenyl ring to the bi-thiophene unit (moving from **1** to **5** and from **4** to **6**), or else by the replacement of the lateral acceptor unit from methyl-pyridinium to methyl-quinolinium (from **2** to **3**).

However, lipophilicity is not the only parameter responsible for the particular localization of each molecule. As a matter of fact, once in the nuclei, the different affinity of the dyes for DNA and RNA determines whether the molecule prefers to localize in the nucleoli or it remains spread in the nucleoplasm. As is the case with **3**, a markedly greater affinity for RNA causes the dye accumulation in the RNA-rich nucleoli, whereas **5** and **6** can be seen diffused in the nuclei as DNA-complexes. Another key aspect is the cell-dependent behavior of the dyes. Broadly speaking, they exert stronger antiproliferative activity against HeLa cells rather than A549 cells, the localization of toxic molecules being better defined in the nuclei of the former. This might be related to the chromosome abundance of HeLa cells, which are typically hypertriploid and thus possess an almost double concentration of nucleic acids that can attract more quantities of dyes for their complexation to occur [51]. 

In light of this, this series of molecules, which had already proved to be interesting for their solvatochromic and non-linear optical properties [36], turned out to be potential antitumor drugs and versatile cellular fluorescent probes, some of which were able to stain specific cell organelles, e.g., endoplasmic reticulum or nucleoli, and others to mark the nuclei, matching the emission of the well-known DAPI dye. In particular, compound **6**, owing to the combined effect of highly conjugated units (bi-thiophene and pyrimidin-pyridinium), resulted in being extremely fascinating for being remarkably fluorescent in the far-red, with a broad emission spectrum peaking at 700 nm and extending over 800 nm. In addition, its fluorescence becomes exceptionally high (Φ_F_ > 0.50), following an explicit interaction with DNA, whose complex is both more favorable than that with RNA (K_ct-DNA/_K_tRNA_ = 7.9) and much more emissive (Φ_F ct-DNA complex_/Φ_F tRNA complex_ = 30), making **6** a specific stain of DNA-rich regions. This compound can thus represent a valuable substitute for DAPI, which is lacking when a nuclear fluorescent probe in the far-red is needed for studying blue-emitting fluorophores. Finally, by virtue of their two-photon absorption abilities, this bicationic series of dyes could also be screened for possible applications in two-photon excited fluorescence microscopy, especially compound **6** which proved to possess the highest two-photon absorption cross-section in the series (hundreds of Goeppert–Mayer, GM) [36]. 

## 4. Materials and Methods

### 4.1. Materials

The investigated compounds **1–6** were synthesized for previous studies [24,36]. All the compounds were dissolved in DMSO of spectroscopic grade (Sigma-Aldrich, Saint Louis, MO, USA) to prepare concentrated stock solutions to be later diluted in water buffers. Spectral and photophysical measurements were carried out in ETN (1 mM EDTA, 10 mM Tris-HCl, 10 mM NaCl) aqueous buffer solutions, pH 7.4. Ethylenediaminetetraacetic acid (EDTA), tris(hydroxymethyl)aminomethane hydrochloride (Tris-HCl), and NaCl were purchased from Sigma-Aldrich.

Baker’s yeast tRNA was purchased from Roche and calf thymus (ct)-DNA from Sigma-Aldrich; they were used after dissolution in sterile ETN. The ct-DNA was additionally sonicated and filtered through a 0.45 µm filter. The concentration of the polynucleotides’ stock solutions was determined spectrophotometrically by recording the incremental absorbance at 258 nm for tRNA (ε = 6900 M^−1^ cm^−1^) and 260 nm and for ct-DNA (ε = 6600 M^−1^ cm^−1^). 

Dulbecco’s modified Eagle’s medium (DMEM), fetal bovine serum (FBS), Trypsin, and Penicillin/Streptomycin were purchased from Euroclone (Pero, Italy). Dimethyl sulfoxide (DMSO) for biological experiments, Trypan Blue powder, and 3-(4,5-dimethylthiazol-2-yl)-2,5-diphenyltetrazolium bromide (MTT) were purchased from Sigma-Aldrich (Saint Louis, MO, USA) and Becton, Dickinson and Company (Franklin Lakes, NJ, USA). 

### 4.2. Spectroscopic Titrations 

Absorption spectra were measured with a Cary 4E (Varian) spectrophotometer choosing a spectral bandpass of 2 nm and using 1 cm path length cuvettes. Fluorescence spectra, recorded as a signal/reference ratio to be independent of excitation intensity fluctuations with appropriate corrections for the instrumental response, were detected by a FluoroMax-4P spectrofluorimeter (HORIBA Scientific) operated by FluorEssence. The path length of the cuvette was 1 cm, and the acquisition of the spectra was conducted in right-angle geometry. The excitation wavelength was chosen as the maximum of the absorption spectrum of the free molecule (A_λexc_ < 0.15). The spectral bandpass was always set at 2 nm for the excitation monochromator, while in emission the spectral bandpass ranged from 10 to 20 nm depending on the system under investigation. Neutral grey filters were put before the samples to dampen the intensity of the excitation light and avoid the photoisomerization of the studied molecules subject to repeated irradiation.

All spectrophotometric and fluorimetric measurements were conducted in ETN aqueous buffer solutions, pH = 7.4, prepared by diluting concentrated stock solutions of compounds **1–6** (DMSO, c = 1.00 mM) in the buffer, allowing to reach a concentration in the micromolar range (0.6–6 µM) for all the six compounds and keep the final DMSO concentration (*v*/*v*) < 2%. Titrations were then performed by adding increasing volumes of nucleic acid stock solutions (c ≈ 2 mM) into the cuvette containing the solution of the studied molecules. The initial volume was 2 mL, and the total volume of nucleic acids added at the end of the titration was 1 mL. After mixing polynucleotides with the investigated compounds at every addition, it was observed that equilibrium was quickly reached in less than 120 s, allowing the recording of the spectra after waiting a standard time of 5 min. The saturation and establishment of a dominant mode of binding were reached in excess of tRNA or ct-DNA r ≤ 0.01 (r = [compound]/[nucleic acid]). Each absorption spectrum was multiplied for the relative dilution factor, while emission spectra were corrected by taking into account the changes in absorbance at the excitation wavelength after every addition. Fluorescence data were processed by means of non-linear fitting to the Scatchard equation (McGhee–von Hippel formalism) [37], which gave values of the ratio of n = [bound compound]/[polynucleotide phosphates] in the range of 0.1–0.2, but for easier comparison, all Ks values were re-calculated at fixed n = 0.2. Only in the case of the first equilibrium of compound **6** with tRNA, the fitting gave an n value equal to 1. The calculated values for Ks have satisfactory correlation coefficients (> 0.99) in all cases. Fluorescence quantum yields of the free ligands (Φ_F_, uncertainty: ±10%) had already been determined rigorously through a comparative method using tetracene in cyclohexane (Φ_F_ = 0.17) and rhodamine 6G in ethanol (Φ_F_ = 0.94) as reference compounds [36]. The determination of Φ_F_ for the tRNA/compound or ct-DNA/compound complexes was here conducted by comparison of the emission spectra of the free ligand (Area_F,free ligand_), used as internal standard, and that of the bound molecule recorded in excess of nucleic acid at the end of the titration (Area_F,complex_) corrected for the fraction of absorbed light: $\Phi_{F,complex}=\frac{Area_{F,\;complex}}{Area_{F,\;free\;ligand}}\times \Phi_{F,free\;ligand}$.

### 4.3. TA Measurements

Ultrafast time-resolved transient absorption measurements were carried out by using a Helios setup, already described elsewhere [52,53]. Femtosecond excitation pulses at 400 nm of ca. 40 fs were generated by using an amplified Ti:sapphire laser system. The pump pulses were passed through a chopper that cut out every second pulse and collimated to the sample in a 2 mm quartz cuvette. Probe pulses for optical measurements were produced by passing a small portion of 800 nm light to an optical delay line with a time window of 3200 ps and focusing it into a 2 mm thick sapphire crystal to generate a white-light continuum in the 450–800 nm range. The white light was focused onto the sample and the differential absorbance (ΔA) in the presence and absence of pump excitation was revealed by a CCD detector at each delay. All the measurements were carried out under magic-angle conditions. Solutions of compounds **1–6** in the presence of tRNA and ct-DNA were prepared by diluting concentrated stock solutions (DMSO, c = 1.00 mM) at a 1/100 dilution ratio. Dilutions were performed in ETN buffer, pH 7.4, containing nucleic acids at a concentration of about 2 mM, to reach r ([compound]/[nucleic acid]) = 0.005 and assure that most of the ligand would be in the bound form. To avoid photoproduct interferences, the solutions were stirred during the experiments and photodegradation was checked by recording the absorption spectrum of the samples before and after the measurements. Transient absorption data were analyzed using Surface Xplorer PRO (Ultrafast Systems) software, which allows performing singular value decomposition of the 3D surface into principal components (spectra and kinetics) and global analysis (giving lifetimes and Decay Associated Spectra (DAS) of the detected transients) [40]. Evolution Associated Spectra (EAS) were calculated performing global analysis according to a consecutive model by means of GloTarAn software [41].

### 4.4. Cell Culture

A549 human alveolar basal epithelial and HeLa (CCL-2™) human cervical epithelial adenocarcinoma cells (ATCC, Manassas, VA, USA) were cultured in a DMEM medium containing 10% (*v*/*v*) heat-inactivated FBS and Penicillin 10,000 U per mL/Streptomycin 10 mg per mL and incubated in a humidified atmosphere (5% CO_2_ at 37 °C). The cell concentration was monitored by Trypan blue dye staining, using an automated cell counter (Invitrogen™ Countess™, Thermo Fisher Scientific, Waltham, MA, USA).

### 4.5. Antiproliferative Assay

A549 and HeLa cells were seeded in Falcon^®^ 96-well clear flat-bottom microplates (Becton, Dickinson and Company, Franklin Lakes, NJ, USA) (2 × 10^3^ cells/well) with 200 µL of DMEM medium. The following day, the medium was replaced with 198 µL of fresh medium adding 2 µL of different dilutions of stock solutions (10 mM) of compounds **1–6** in DMSO (% DMSO = 1), to reach final concentrations ranging from 100 to 0.01 μM in quadruplicate. In each experiment, two quadruplets were kept as control (200 µL of DMEM medium) and two other quadruplets were used as vehicle controls to take into account the contribution of DMSO (198 µL of DMEM medium + 2 µL of DMSO). After 72 h of incubation under standard culture conditions, the cells were treated with 20 µL of a 5 mg/mL 3-(4,5-Dimethylthiazol-2-yl)-2,5-Diphenyltetrazolium Bromide (MTT) dye solution to reach a concentration of 0.5 mg/mL in the wells. The cells were then incubated under the usual conditions for 3 h to allow the formation of formazan crystals. Dark blue formazan crystals were then solubilized in 150 µL DMSO at 37 °C for 1 h, and the corresponding absorbance at 570 nm was measured using a microplate reader (Beckman Coulter DTX880, Beckman Coulter, Inc., Brea, CA, USA) after a brief mechanical shaking. Cell viability was expressed as the 570-nm absorbance percentage reduction in treated cells compared to controls, assuming the absorbance of controls was 100% [(absorbance of treated wells/absorbance of control wells) × 100]. All measurements were performed during three independent experiments, and the results are reported as average values ± SD. The comparison between the proliferation of the cells treated with the dyes and those grown without external agents (controls) is expressed in terms of half maximal inhibitory concentration IC_50_: the concentration of the compound that induces 50% cell growth inhibition.

### 4.6. Fluorescence Microscopy

3 × 10^3^ A549 or HeLa cells were seeded on round glass coverslips previously sterilized by 30 s of immersion in 70% ethanol, rinsed with sterile phosphate buffer saline (PBS), and placed in a Falcon^®^ 24-well clear flat-bottom multiwell cell culture plates (Becton, Dickinson and Company, Franklin Lakes, NJ, USA). The cells were then incubated for 45 min in a humidified atmosphere (5% CO_2_ at 37 °C) and, subsequently, 500 µL of DMEM medium were gently added to each well. After that, cells were incubated for 24 h under standard culture conditions (humidified atmosphere with 5% CO_2_ at 37 °C). In addition, 400 µL of a solution containing compounds **1–6** diluted in DMEM at the final concentration of 10 μM were then administered to the cells, and the multiwell was again incubated for 2 h under canonical conditions. Cells on round glass coverslips were then rinsed twice with PBS and fixed in 4% paraformaldehyde for 20 min at room temperature. After washing with PBS, samples were mounted and nuclei were stained with Vectashield^®^ Vibrance™ Antifade Mounting Medium containing 4′,6-diamidino-2-phenylindole (DAPI) (Vector Laboratories Inc., Burlingame, CA, USA) [54]. Image acquisition was performed by using a fluorescence microscope (Eclipse TE2000-S, Nikon, Tokyo, Japan) equipped with the F-View II FireWire camera (Olympus Soft Imaging Solutions GmbH, Münster, Germany) and through the use of Cell^F^ Imaging Software (Olympus Soft Imaging Solutions GmbH, Münster, Germany). The photostability of the six compounds was checked by evaluating the differential pixel density of images taken at t = 0 and after 5-min exposure to constant irradiation from the microscope excitation lamp.

## 5. Conclusions

Six water-soluble bications with important emissions in aqueous media, spanning the visible spectrum up to the sought-after far-red spectral region, were investigated as for their interaction with nucleic acids and their structure–property relationships, on which their bioavailability and bioactivity depend.

In most cases, a gain in the fluorescence efficiency was found for the studied bications when linked to DNA and/or RNA, suggesting these molecules as potential “light-up” bioprobes. This study also proved how small structural modifications (replacing the central dimethoxy phenyl ring with the bi-thiophene unit, or the lateral methyl-pyridinium groups with the methyl-quinolinium ones) could tune the amphiphilicity of these bications, leading to very distinct bioactivity in terms of both biological response and intracellular localization. Particularly, the most lipophilic bications, likely the most permeant, were found to exert the highest antiproliferative activity. These promising anticancer therapeutic agents clearly stained the cellular nucleus with a long-wavelength bright emission, offering a red alternative to DAPI, as evidenced by fluorescence microscopy, which indeed provided also further proof of direct cytotoxicity. On the other hand, less amphiphilic compounds, preferentially localized in the cytoplasm and in a unique case in a more specific cellular compartment, presumably the endoplasmic reticulum, could be considered appealing candidates as non-toxic fluorescent biomarkers.

## Figures and Tables

**Table 1:** Spectral properties (absorption maxima, λ_abs_, with the main absorption maximum underlined, emission maxima, λ_em_, and Stokes shifts, Δν) and fluorescence quantum yields (Φ_F_) of compounds **1–6** (free in water, W, with 5% DMSO) and their complexes with tRNA and ct-DNA (with the molar ratio, r = [compound]/[nucleic acid], reached in each titrations) in ETN buffer solution. Fluorescence quantum efficiencies of the complexes were calculated from the ratio of fluorescence intensities of a free and bound ligand.

Comp.	W				tRNA					ct-DNA				
	λ_abs_/ nm	λ_em_/ nm	Δν/ cm^−1^	Φ_F_	λ_abs_/ nm	λ_em_/nm	Φ_F_	Δν/ cm^−1^	r	λ_abs_/ nm	λ_em_/ nm	Φ_F_	Δν/ cm^−1^	r
**1**	352, 427	583	6270	0.16	368, 467	577	0.025	4080	0.0052	365, 464	578	0.084	4250	0.0075
**2**	359, 446	605	5890	0.36	372, 471 377, 488	600 595	0.066 0.12	4560 3690	0.081 0.0027	374, 481	591	0.17	3870	0.0081
**3**	393, 485	680	5910	0.010	407, 524	655	0.028	3820	0.0028	405, 520	645	0.058	3730	0.0050
**4**	389, 498	710	6000	0.003	408, 543	680	0.0068	3710	0.0025	405, 532	670	0.012	3870	0.0040
**5**	464	600	4890	0.026	495	615	0.057	3940	0.0036	486	595	0.10	3770	0.0025
**6**	540	700	4230	0.058	500 571	665 705	0.002 0.018	4960 3330	0.63 0.0028	567	700	0.56	3350	0.0017

**Table 2:** Binding constants (logK) of compounds **1–6** with tRNA and ct-DNA calculated by processing fluorimetric titrations.

Compound	tRNA	ct-DNA
	logK	logK
**1**	5.49 ± 0.06	7.11 ± 0.04
**2**	6.4 ± 0.2 4.3 ± 0.5	6.85 ± 0.04
**3**	5.2 ± 0.1	4.66 ± 0.09
**4**	4.4 ± 0.3	4.5 ± 0.1
**5**	5.19 ± 0.07	5.0 ± 0.1
**6**	8.0 ± 0.8 4.4 ± 0.2	5.3 ± 0.3

**Table 3:** Excited-state properties of compounds **1–6** in water (W) with 5% DMSO, complexed with tRNA and ct-DNA [r ([compound]/[nucleic acid]) = 0.005] in ETN buffer solution, obtained by ultrafast transient absorption (TA) (λ_exc_ = 400 nm).

Compound	Free (W) ^a^	tRNA	ct-DNA	Assignment
	τ (ps)			
**1 ^b^**	0.79	0.74	2.0	Solv.
	6.1			Solv./VR
		8.0	8.1	S_1_ (Complex)
	190	72	51	CR
			1100	S_1_ (Complex)
	2700			S_1_ (Free)
		1600	3500	S_1_ (Complex)
**2 ^b^**	0.60	0.13		Solv.
	1.1		1.7	Solv.
		5.2	12	Solv./VR
		57	68	S_1_ (Complex)
	190			CR
	1500		1200	S_1_ (Free)
		1200	4500	S_1_ (Complex)
**3**	0.22			Solv.
	1.7	2.2	2.6	Solv.
	23	32	57	CR
	110			S_1_ (Free)
		300	770	S_1_ (Complex)
**4**	0.90	0.78		Solv.
	3.3	4.4	2.1	Solv.
		25	20	CR
	39			S_1_ (Free)
		240	270	S_1_ (Complex)
	Inf			T_1_
**5**	0.66			Solv.
	4.9	2.6	2.9	Solv.
		38	55	CR
	92			S_1_ (Free)
		300	310	S_1_ (Complex)
	Inf	Inf	Inf	T_1_
**6**	0.53	1.2		Solv.
	19		7.0	Solv./VR
	160	60	330	CR
	910			S_1_ (Free)
		260	3600	S_1_ (Complex)
		Inf		T_1_

^a^ Data in water (5% DMSO) retrieved from [36]. ^b^ Data referring to the complexation with ct-DNA (r = 0.05) retrieved from [24].

**Table 4:** Tumor cell growth inhibition presented as IC_50_ values (in μM) evaluated from the MTT test after 72-h incubation.

	Compound	IC_50_/µM					
Cell		1	2	3	4	5	6
A549		-	>100	12 ± 2	-	27 ± 9	19 ± 5
HeLa		-	53 ± 2	1.7 ± 0.1	13 ± 2	18 ± 6	0.7 ± 0.2

## Data Availability

Data is contained within the article or Supplementary Material.

## Supplementary Materials

The following supporting information can be downloaded at: https://www.mdpi.com/article/10.3390/molecules27123713/s1, Figure S1: spectrometric titrations of compounds **2**, **3,** and **6** with tRNA; Figures S2–S6: quantitative absorption spectra of compounds **1–6** when bound to nucleic acids; Figures S7–S11: fluorimetric titrations of compounds **1–6** with nucleic acids; Figures S12 and S13: Ultrafast transient absorption experiments of compounds **1–4** with nucleic acids; Figure S14: Antiproliferative effect of compounds **1–6**; Figures S15 and S16: Fluorescence microscopy images of different cell lines loaded with compounds **1–6**; Table S1: Consensus LogP values of compounds **1–6**.

## Sample Availability

Samples of the compounds **1–6** are available from the authors.
