# Supplementary material for: Amphiphilicity-Controlled Localization of Red Emitting Bicationic Fluorophores in Tumor Cells Acting as Bio-Probes and Anticancer Drugs
Source: Molecules. 2022 Jun 9;27(12):3713. doi: 10.3390/molecules27123713 (PMC9230006; doi:10.3390/molecules27123713)
Supplement: Supplementary file 1 [file molecules-27-03713-s001.zip › molecules-1747494-supplementary.pdf]

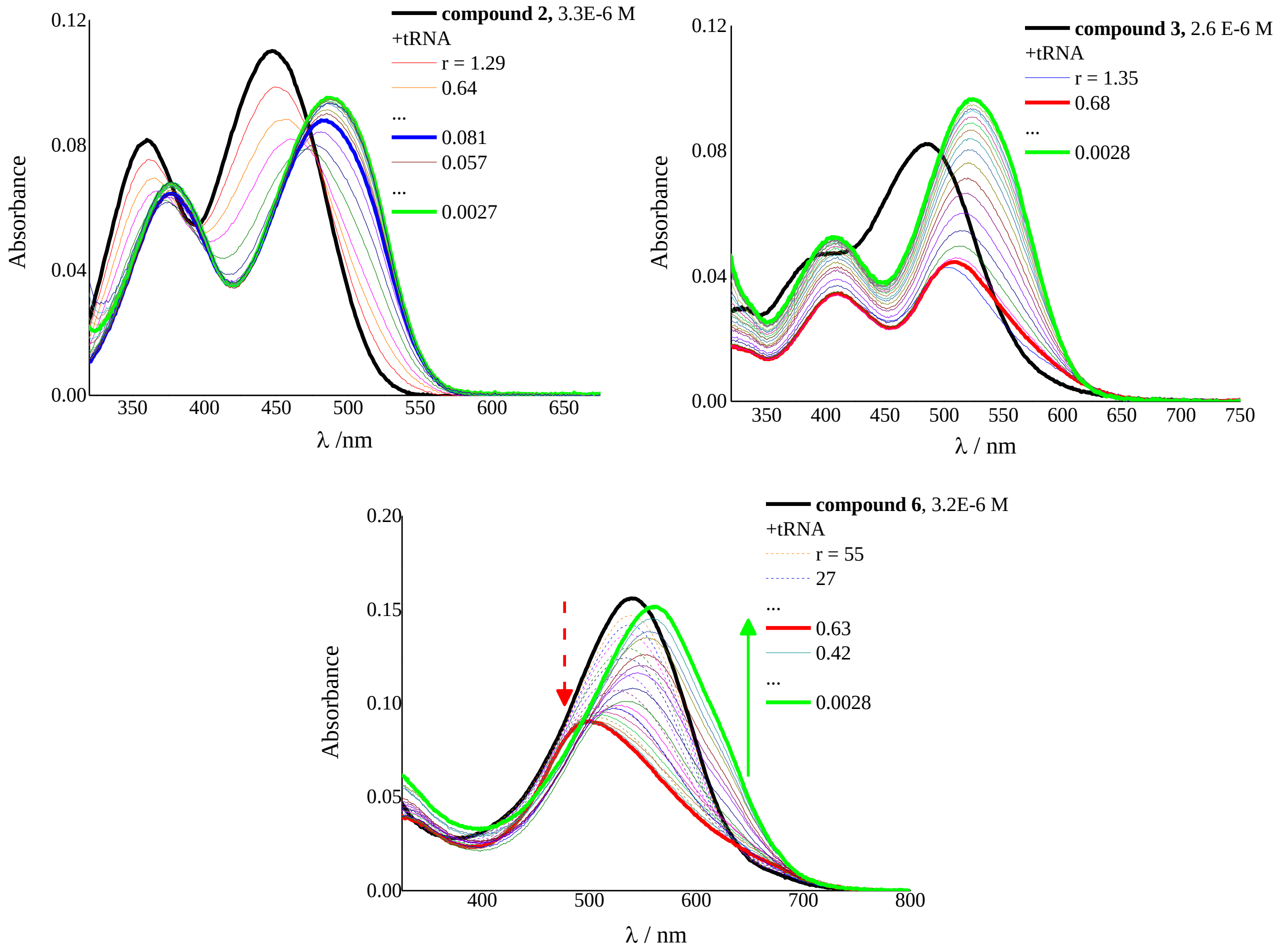

Figure S1. Changes in the absorption spectrum of **2** (c = 3.3 μM), **3** (c = 2.6 μM) and **6** (c = 3.2 μM) in ETN buffer solution, pH 7.4, upon titration with tRNA.

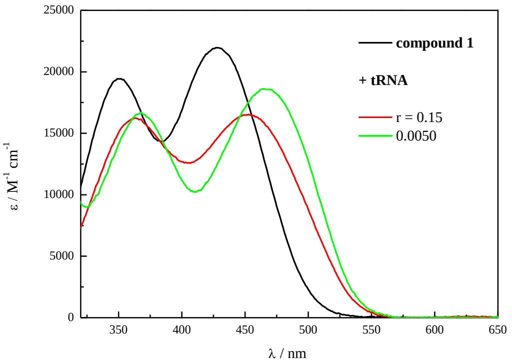

Figure S2. Quantitative absorption spectra of **1** and its complexes with tRNA in ETN buffer solution, pH 7.4.

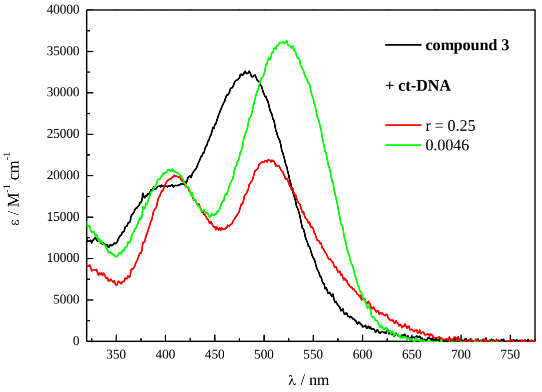

Figure S3. Quantitative absorption spectra of **3** and its complexes with ct-DNA in ETN buffer solution, pH 7.4.

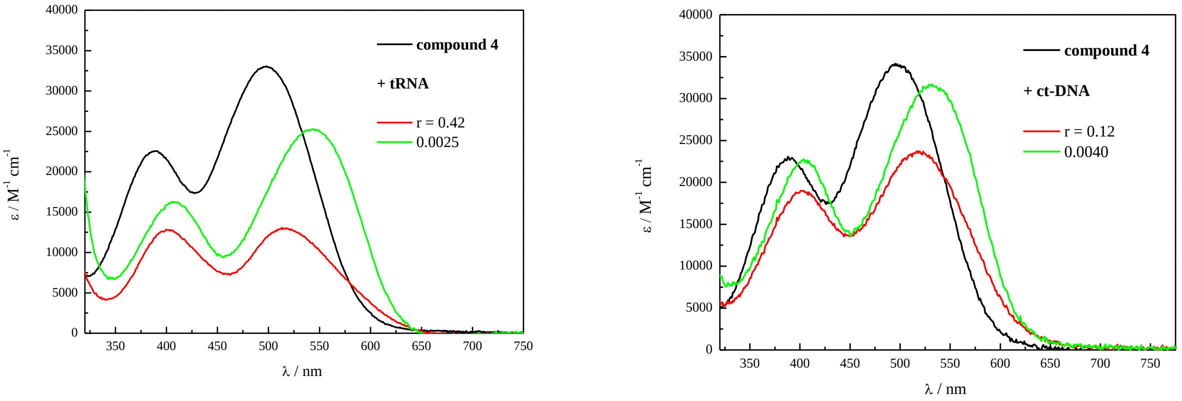

Figure S4. Quantitative absorption spectra of **4** and its complexes with tRNA (LEFT) and ct-DNA (RIGHT) in ETN buffer solution, pH 7.4.

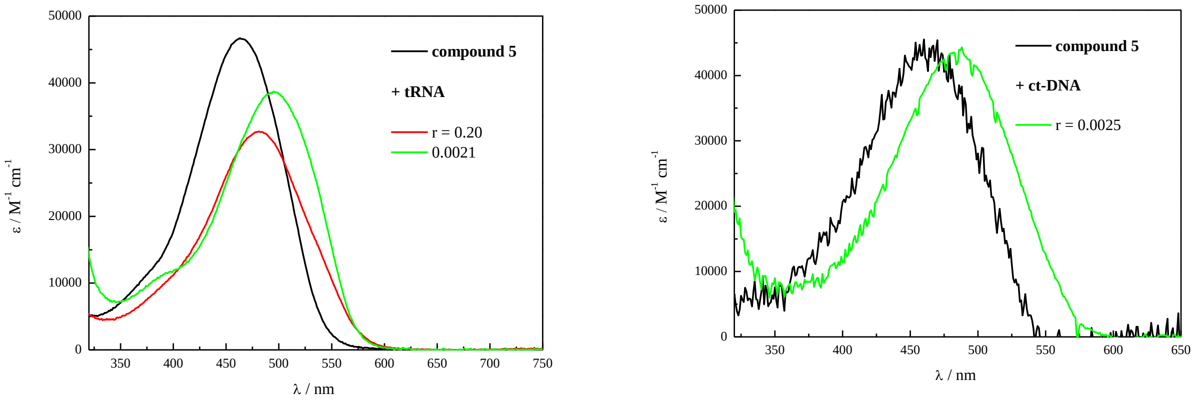

Figure S5. Quantitative absorption spectra of **5** and its complexes with tRNA (LEFT) and ct-DNA (RIGHT) in ETN buffer solution, pH 7.4.

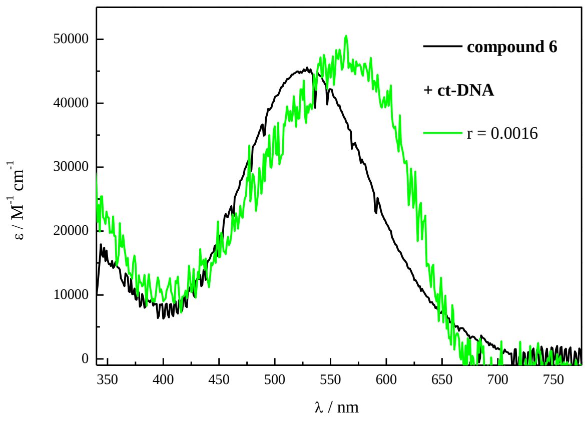

Figure S6. Quantitative absorption spectra of **6** and its complexes with ct-DNA in ETN buffer solution, pH 7.4.

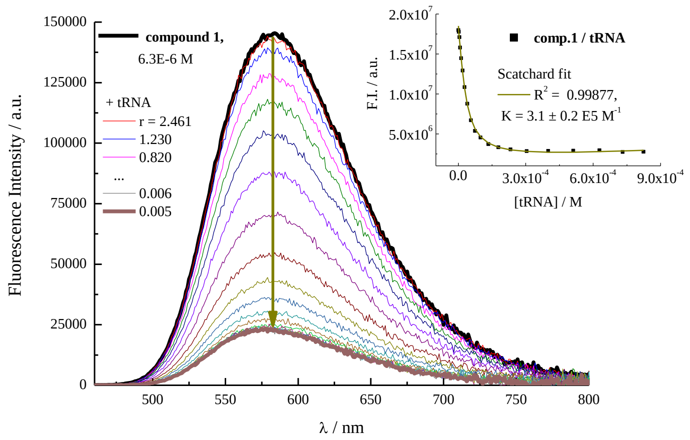

Figure S7. Changes in the fluorescence spectrum of **1** (λ_{exc} = 430 nm, c = 6.3 μM) in ETN buffer solution, pH 7.4, upon titration with tRNA; Inset: fluorescence intensity of **1** as a function of tRNA concentration and its fitting according to the Scatchard equation (n = 0.2).

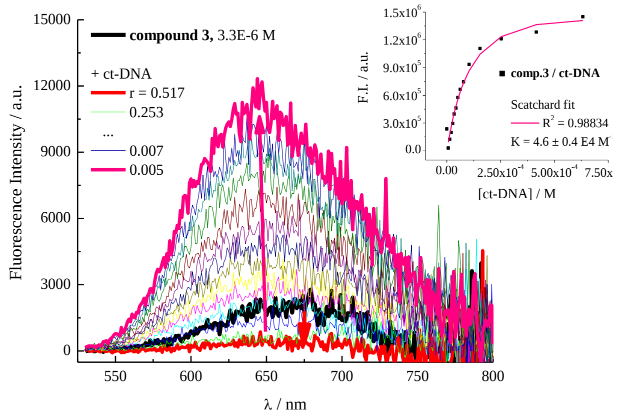

Figure S8. Changes in the fluorescence spectrum of **3** (λ_{exc} = 500 nm, c = 3.3 μM) in ETN buffer solution, pH 7.4, upon titration with ct-DNA; Inset: fluorescence intensity of **3** as a function of ct-DNA concentration and its fitting according to the Scatchard equation (n = 0.2).

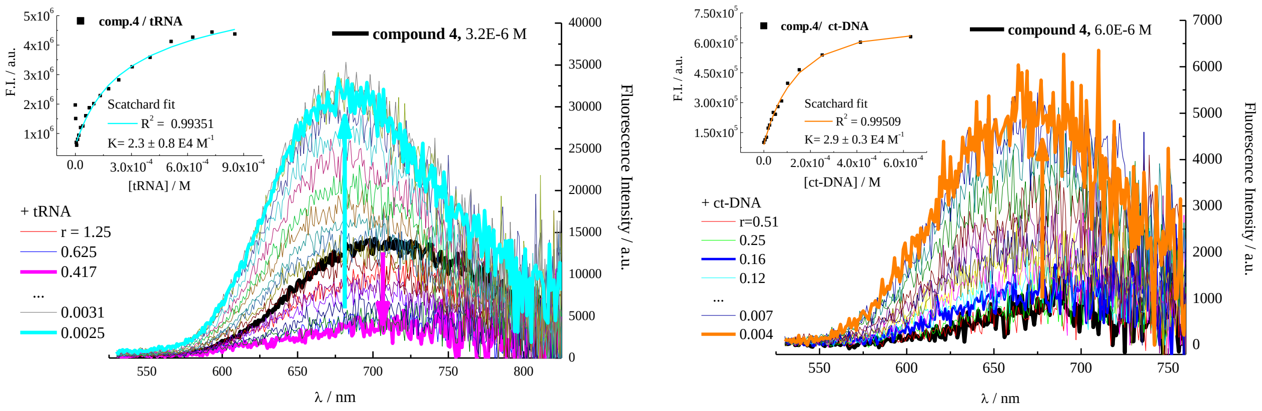

Figure S9. Changes in the fluorescence spectrum of **4** in ETN buffer solution, pH 7.4, upon titration with tRNA (Left, λ_{exc} = 500 nm, c = 3.2 μM) and ct-DNA (Right, λ_{exc} = 500 nm, c = 6.0 μM); Insets: fluorescence intensities of **4** as a function of tRNA and ct-DNA concentration and their fitting according to the Scatchard equation (n = 0.2).

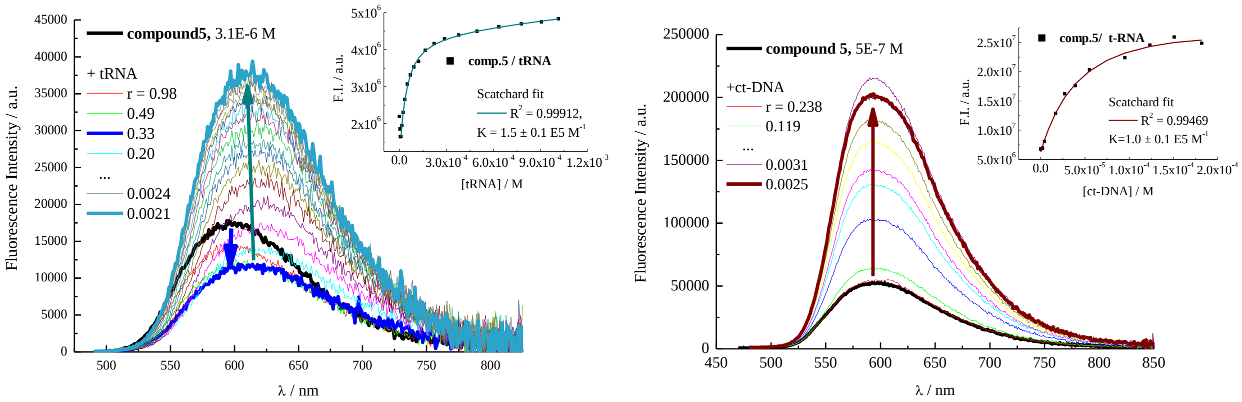

Figure S10. Changes in the fluorescence spectrum of **5** in ETN buffer solution, pH 7.4, upon titration with tRNA (Left, λ_{exc} = 465 nm, c = 3.1 μM) and ct-DNA (Right, λ_{exc} = 468 nm, c = 0.5 μM); Insets: fluorescence intensities of **5** as a function of tRNA and ct-DNA concentration and their fitting according to the Scatchard equation (n = 0.2).

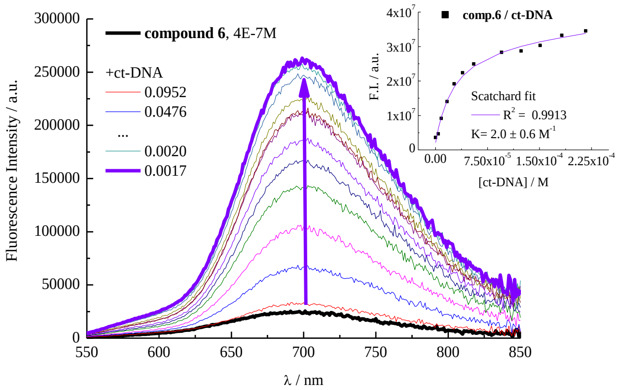

Figure S11. Changes in the fluorescence spectrum of **6** (λ_{exc} = 522 nm, c = 0.4 μM) in ETN buffer solution, pH 7.4, upon titration with ct-DNA; Inset: fluorescence intensity of **6** as a function of ct-DNA concentration and its fitting according to the Scatchard equation (n = 0.2).

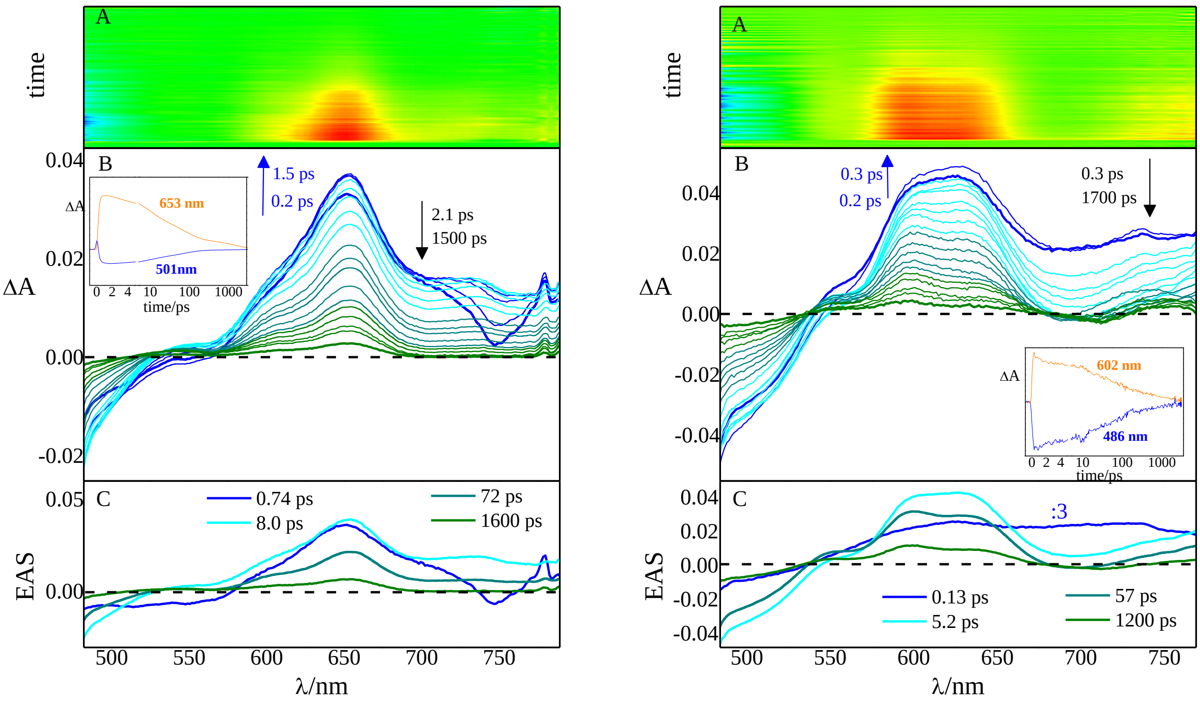

Figure S12. Pump–probe absorption spectroscopy of compounds **1** (Left graph) and **2** (Right graph) in ETN buffer solution, pH 7.4, complexed with tRNA, r ([compound]/[tRNA]) = 0.005 (λ_{exc} = 400 nm): (A) contour plot of the experimental data, (B) time-resolved absorption spectra recorded at different delays after the laser pulse (inset: decay kinetics recorded at meaningful wavelengths), and (C) Evolution Associated Spectra (EAS) obtained by global analysis.

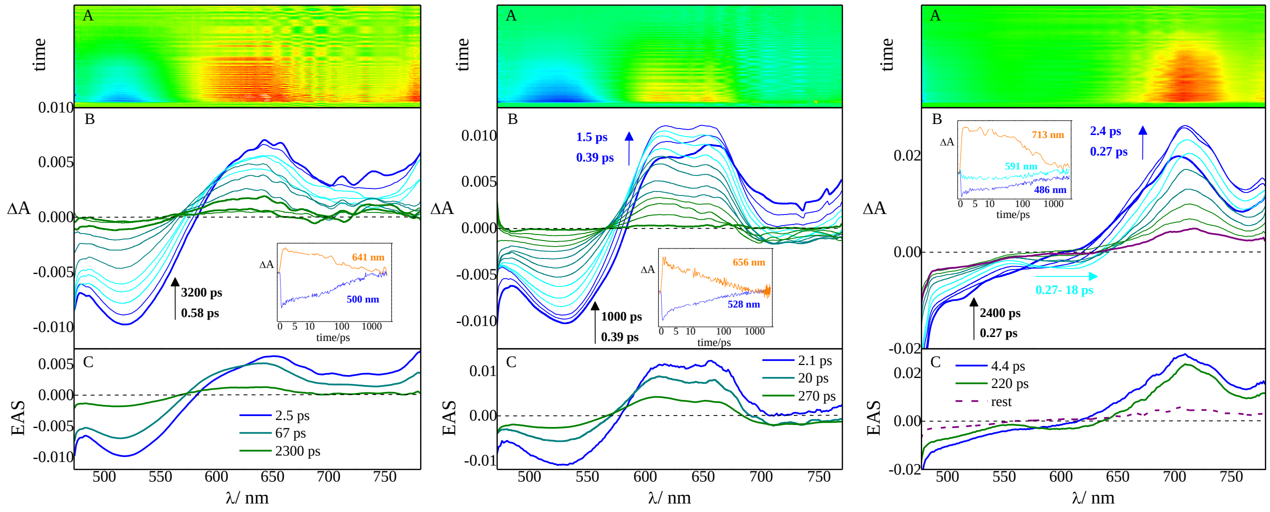

Figure S13. Pump–probe absorption spectroscopy of compounds **3** (Left graph), **4** (Central graph), and **5** (Right graph) in ETN buffer solution, pH 7.4, complexed with ct-DNA, r ([compound]/[ct-DNA]) = 0.005 (λ_{exc} = 400 nm): (A) contour plot of the experimental data, (B) time-resolved absorption spectra recorded at

different delays after the laser pulse (inset: decay kinetics recorded at meaningful wavelengths), and (C) Evolution Associated Spectra (EAS) obtained by global analysis.

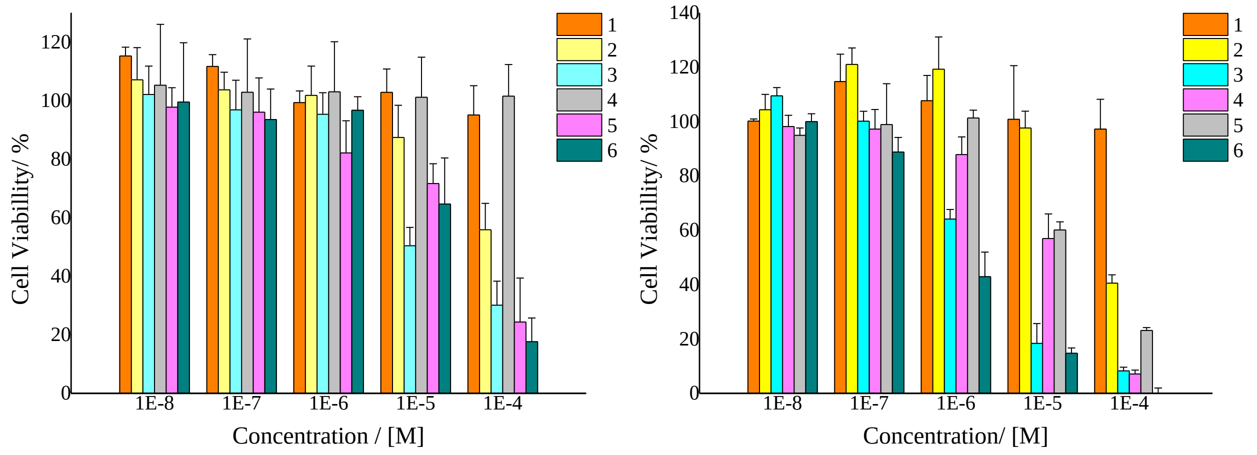

Figure S14. Antiproliferative effect exerted by compounds **1-6** on A549 (Left) and HeLa (Right) cell lines, expressed as mean of two independent experiments of four replicas each ± SD.

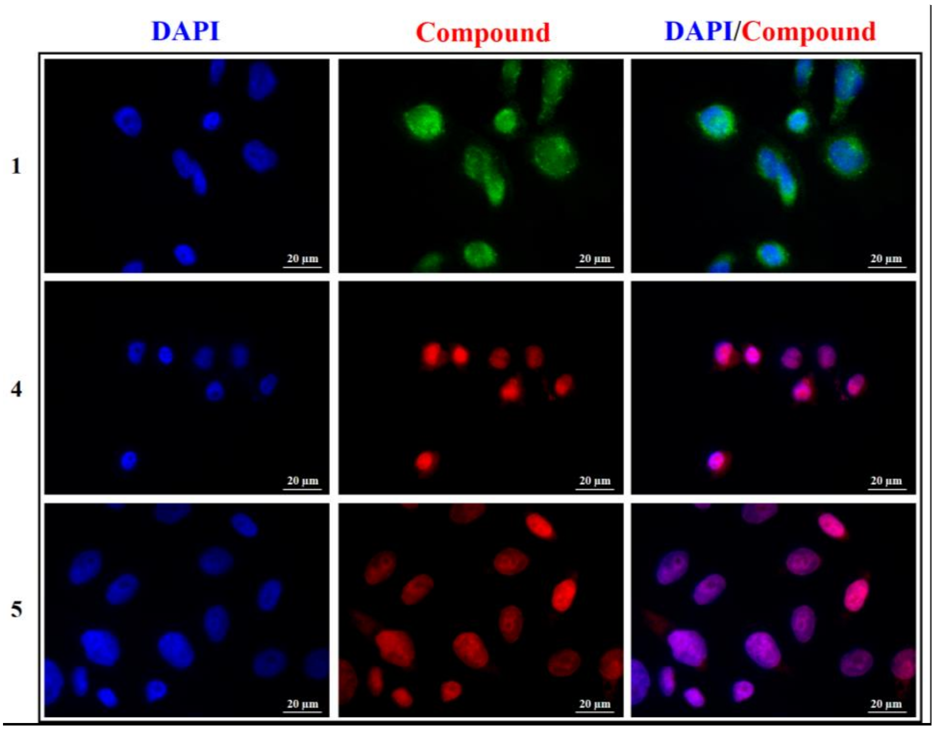

Figure S15. Representative fluorescence microscopy images of nuclei (DAPI, blue), compound **1** (green), and compounds **4** and **5** (red), and the relative merged images of HeLa cells. Image magnification: 60 ×.

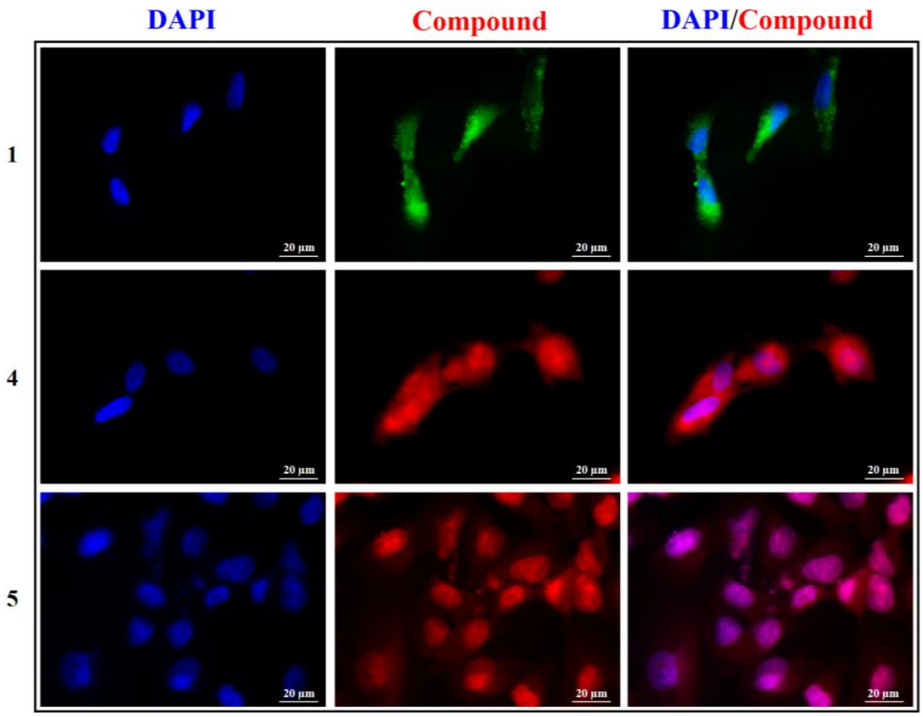

Figure S16. Representative fluorescence microscopy images of nuclei (DAPI, blue), compound **1** (green), and compounds **4** and **5** (red), and the relative merged images of A549 cells. Image magnification: 60 ×.

Table S1. cLogP (consensus LogP) descriptor of compounds **1-6** calculated using the SwissADME web tool.

**Compounds**	**1**	**2**	**3**	**4**	**5**	**6**
**cLogP**	3.33	3.35	4.91	3.50	4.69	4.86
